# Inhibition of Murine Breast Cancer Metastases by Hydrophilic As_4_S_4_ Nanoparticles Is Associated With Decreased ROS and HIF-1α Downregulation

**DOI:** 10.3389/fonc.2019.00333

**Published:** 2019-04-26

**Authors:** Tao Wang, Jie Meng, Chuan Wang, Tao Wen, Mengfan Jia, Yangyang Ge, Lifei Xie, Suisui Hao, Jian Liu, Haiyan Xu

**Affiliations:** Institute of Basic Medical Sciences, Chinese Academy of Medical Sciences & Peking Union Medical College, Beijing, China

**Keywords:** breast cancer, As_4_S_4_ nanoparticles, reactive oxygen species, hypoxic tumor microenvironment, inflammation

## Abstract

Arsenic sulfide (As_4_S_4_) is a mineral drug that can be administrated orally and has been applied in the treatment of myeloid leukemia. The aim of this work is to investigate the therapeutic effect of As_4_S_4_ in highly metastatic triple-negative breast cancer (TNBC) animal model, as As_4_S_4_ has not been applied in the treatment of breast cancer yet. To overcome the poor solubility of original As_4_S_4_, a formulation of hydrophilic As_4_S_4_ nanoparticles (e-As_4_S_4_) developed previously was applied to mouse breast cancer cells as well as the tumor-bearing mice. It was shown that e-As_4_S_4_ was much more cytotoxic than r-As_4_S_4_, strongly inhibiting the proliferation of the cells and scavenging intracellular reactive oxygen species (ROS). The oral administration of e-As_4_S_4_ significantly increased the accumulation of arsenic in the tumor tissue and eliminated ROS in tumor tissues. Besides, e-As_4_S_4_ could also inhibit the activation of hypoxia-inducible factor-1α (HIF-1α) and NLRP3 inflammasomes. Consequently, the angiogenesis was reduced, the metastasis to lung and liver was inhibited and the survival of tumor-bearing mice was prolonged. In conclusion, e-As_4_S_4_ holds great potential for an alternative therapeutics in the treatment of breast cancer, due to its unique function of correcting the aggressive microenvironment.

## Introduction

Metastasis is the leading cause of breast cancer mortality, which has been one major challenge in clinical treatment (1). In particular, triple-negative breast cancer (TNBC) is characterized by the absence of estrogen receptors (ER), progesterone receptors (PR) and HER2 receptors, which is one of the most aggressive types of breast cancers, marked by high rates of relapse, visceral metastases and early death (2, 3). The treatment of TNBC is local therapy with surgery and/or radiotherapy followed by standard anthracycline and/or taxane-based systemic adjuvant treatment (3, 4). Despite an initial response to systemic chemotherapy, patients with TNBC often follow an aggressive course of progression and metastasis and develop multidrug resistance to conventional therapies (5). Besides, patients with TNBC are less sensitive to typical endocrine therapies due to lack of the receptors (6). Therefore, there is still an unmet need in the treatment of breast cancer, and the development of new therapeutics for preventing and treating metastasis for the management of TNBC patients is of great significance.

Arsenic sulfide (As_4_S_4_, or termed as realgar) is a mineral drug that can be orally administrated and has shown a certain therapeutic effect in acute promyelocytic leukemia (APL) (7) and chronic myeloid leukemia (CML) (8) in clinics. Nevertheless, the poor solubility in water and the acidic solution of raw As_4_S_4_ (r-As_4_S_4_) leads to the extremely low bioavailability (9). In recent years we and other research groups have made efforts to increase its poor water solubility by reducing the original crystal size into nanoscale in different ways (10–12). We have demonstrated that the bioavailability and the therapeutic effect of As_4_S_4_ were increased significantly on acute myeloid leukemia (AML) *in vitro* and *in vivo* when the crystal particles were reduced into nanoscale and encapsulated with hydrophilic polymers (11). Moreover, nanoscaled As_4_S_4_ also inhibited the proliferation of multiple myeloma cells (10) and melanoma cancer cells (12) effectively.

Encouraged by these achievements, in this work, we applied the lab-developed formulation of hydrophilic As_4_S_4_ nanoparticles (e-As_4_S_4_) to the TNBC mouse model, investigating the effect of e-As_4_S_4_ in this kind of metastatic solid tumor and unveiling underlying mechanisms. We showed that the oral administration of e-As_4_S_4_ led to arsenic accumulation in the tumor tissue, which reduced angiogenesis and inflammasome in the tumor microenvironment by downregulating the reactive oxygen species (ROS) level, while arsenic hardly accumulated in tumor tissue of r-As_4_S_4_ treated group. As a result, tumor metastasis to lung and liver was inhibited significantly and the survival of breast cancer mice was prolonged as well.

## Materials and Methods

### Reagents

As_4_S_4_ (CID: 3627253) was purchased from Alfa Aesar Co. (Ward Hill, MA). Polyvinyl caprolactam-polyvinyl acetate-polyethylene glycol (PVCL-PVAc-PEG, commercial name: Soluplus; average molecular weight = 1.18 × 10^5^ Da) was provided by BASF SE (Ludwigshafen, Germany). Modified RPMI medium, DMEM/high glucose medium and penicillin-streptomycin solution were purchased from Hyclone (GE healthcare life sciences, Logan, Utah, USA). Fetal bovine serum (FBS) was purchased from Gibco (Life Technologies, Carlsbad, CA, USA). HEPES solution (pH = 7.4) and L-glutamine were purchased from the Cell Resource Center of the Chinese Academy of Medical Sciences (Beijing, China). RIPA cell lysis buffer was purchased from Beyotime Biotechnology (Haimen, China). 2′,7′-Dichlorofluorescein diacetate (DCFH-DA) and cell counting kit (CCK-8) reagent was purchased from Dojindo (Japan). Phenylmethanesulfonyl fluoride (PMSF) was purchased from Sigma-Aldrich (St. Louis, MO, USA). Other chemicals used in this work were of analytical reagent grade and from Beijing Chemical Reagent Company.

### Preparation and Characterization of e-As_4_S_4_

The e-As_4_S_4_ was prepared according to the procedure previously reported (11). The commercial compound As_4_S_4_ (powder of 28 μm in average diameter) and soluplus were mixed in a ratio of 1:15 (w/w) and fed into a co-rotating twin-screw extruder (HAAKE MiniLab II, ThermoFisher Scientific, Rockford, IL). The processing conditions were set as below: blending temperature in the mix chamber 120°C, screw rotation rate 10 rpm and cycling time 70 min. The co-extruded material was ground in a coffee grinder at room temperature; the resulting product was e-As_4_S_4_ for uses in the cellular and *in vivo* experiments. To prepare the suspension for cell culture and animal experiment, the powder of r-As_4_S_4_ or e-As_4_S_4_ was added to saline and sonicated in an ultrasound bath for 10 min at room temperature. The size distribution of e-As_4_S_4_ suspension was measured using dynamic light scattering (DLS, Nano ZS90 Zetasizer, Malvern Instruments, Malvern, UK). In addition, the suspension was dripped on carbon-coated copper grids and air-dried at room temperature for the measurement of scanning transmission electron microscopy (STEM) and energy-dispersive X-ray analysis (EDX) elemental mapping (Tecnai G^2^ F20 U-Twin microscope).

### Cell Culture

Mouse macrophage RAW 264.7 cells were purchased from the Cell Resource Center of the Chinese Academy of Medical Sciences (Beijing, China) and cultured in DMEM/high glucose medium supplemented with 10% FBS, 100 U/mL penicillin and 100 μg/ml streptomycin. Mouse breast cancer cell 4T1 cells were purchased from Cell Bank of Shanghai Institutes of Biological Sciences, Chinese Academy of Sciences (Shanghai, China) and cultured in modified RPMI medium supplemented with 10% FBS, 100 U/ml penicillin, 100 μg/ml streptomycin, 2 mM L-glutamine, and 5 mM HEPES.

### Cell Viability Assay

Cells of 4T1 or RAW 264.7 were incubated with e-As_4_S_4_ or r-As_4_S_4_ at 10, 20, 40, 80, and 160 mg/L of As_4_S_4_ or with fresh medium as a control group. After 48 h incubation, cells were washed twice with PBS, supplemented with 100 μL fresh medium, and then 10 μL CCK-8 reagent was added to each of the wells and incubated at 37°C for 1 h. The absorbance value (optical density, OD) was read at 450 nm and normalized with the control group. Measurements were carried out in quadruplicates.

### Intracellular Reactive Oxygen Species (ROS) Measurement

Intracellular ROS was measured using DCFH-DA. Cells of 4T1 or RAW 264.7 were incubated with e-As_4_S_4_, r-As_4_S_4_, and soluplus, and fresh medium as a control group. After 24 h incubation, cells were washed and further incubated with DCFH-DA at 10 μM at 37°C for 30 min. Afterward, cells were washed by PBS and suspended in 100 μL PBS for flow cytometer analysis. Data were presented as mean ± SD.

### Animal Model and Drug Administration

Six-week-old, female BALB/c mice were bred at the Experimental Animal Center at the Institute of Basic Medical Sciences, Chinese Academy of Medical Sciences (Beijing, China) under specific pathogen-free conditions. All the animal experiments reported herein were carried out in compliance with the regulations of Chinese Academy of Medical Sciences Standing Committee on animal experiments.

To establish the TNBC mouse model, 4T1 cell suspensions in PBS (pH = 7.4) were injected orthotopically close to the nipples, in the mammary fat pad of the naive female BALB/c mice (1 × 10^6^ cells/mouse) in the right flank. Most of the mice developed visible solid tumors (≥4 mm in diameter) on day 7 post-inoculation. The well-established tumor-bearing mice were randomly divided into four groups (*n* = 11) as follows: e-As_4_S_4_, r-As_4_S_4_, soluplus, and saline. Mice in each group were administrated intragastrically. The treatment regime for each group was described in Table 1. After 3 weeks of treatment (day 28 post-inoculation), five or six mice were randomly selected from each group and sacrificed. The tumor tissues, livers and lungs were collected and fixed in 10% neutral buffered formalin. The other mice left in each group continued to receive treatment until death. During the treatment, the tumor size of each mouse was measured every other day.

**Table 1:** Intragastric administration protocols.

**Group**	**Solution (mg/mL)**		**Administration regime**
	**As_**4**_S_**4**_**	**Soluplus**	
e-As_4_S_4_	9	135	200 μL per time, twice per day
r-As_4_S_4_	9	0	
Soluplus	0	135	
Saline	/	/	

### Arsenic Distribution in Organs Measurement

Part of the sacrificed mice organs, including liver, kidney, brain, and tumor tissues were collected and weighted precisely and soaked in digestion solution consisting of high purity nitric acid and hydrogen peroxide (3:1 in volume), followed by incubating in a 100°C water bath for 30 min. The final sample solutions were diluted to 4 mL of deionized water. Atomic fluorescence spectroscopy (AFS, AFS-8230 HG-AFS, Beijing Titan Instruments Co., Ltd., Beijing, China) was applied to examine the arsenic content in the organs and their concentrations in each tissue were calculated. The accumulations of arsenic in these organs were expressed with the relative concentration in per gram of wet tissues (w/w).

### Analysis of ROS in Mice Tumors

Detection of ROS was performed by dihydroethidium (DHE) assay according to the manufacturers' instruction. Briefly, part of the fresh tumors was embedded into optimum cutting temperature (OCT) compound (Sakura, Japan), and cut into 5 μm-thick sections. DHE (10 mol/L, Sigma) was applied to each tissue section and incubated at room temperature for 30 min. After washing, the sections were stained with DAPI (Beyotime) for 10 min and then mounted and coverslipped. the slides were imaged by a fluorescence microscope (Olympus BX53, Japan). All the processes mentioned above should be protected from light. The average integrated option density (IOD) of DHE in 3 randomly selected areas for each group were calculated by Image J.

### Electron Spin Resonance (ESR) Spectroscopic Measurements

All ESR measurements were carried out using a Bruker EMX ESR spectrometer (Billerica, MA) at ambient temperature. Fifty microliter aliquots of sample solution were put in glass capillary tubes with internal diameters of 1 mm and sealed. The spin trap, DMPO, was used to identify superoxide anion and hydroxyl radical during the ESR measurements. The chemical KO_2_ system (superoxide anion was generated by dissolving KO_2_ in DMSO solvent in the presence of crown ether) was used to verify the ability of scavenging. The Fenton reaction system (·OH was generated by reaction of FeSO_4_ and H_2_O_2_) was used to verify the ability of scavenging ·OH. Other settings were as follows: 1 G field modulation, 100 G scan range, and 20 mW microwave power for superoxide anion detection. 2 G field modulation, 100 G scan range, and 10 mW microwave power for hydroxyl radical detection.

### Histological, Immunohistochemical and Immunofluorescence Analysis

Hematoxylin and eosin (H&E) staining, immunohistochemical (IHC) staining and immunofluorescence staining were all conducted using standard techniques (Servicebio Co, China). Briefly, for the IHC, the slides were deparaffinized and antigen retrieval was then performed using a microwave oven in EDTA, pH = 8.0 (Servicebio, Wuhan, China). Primary antibodies of HIF-1α (Abcam, Rabbit IgG polyclonal, Cambridge, UK), CD31 (Servicebio, Rabbit IgG polyclonal), and NLRP3 (Servicebio, Rabbit IgG polyclonal) were applied overnight before HRP-labeled Goat Anti-Rabbit IgG (H+L) (Servicebio) incubation for 50 min at room temperature. DAB was used as chromogens and slides were counterstained with hematoxylin before mounting. For the immunofluorescence staining, primary antibodies for CD31 (Servicebio, Rabbit IgG polyclonal) and α-SMA (Servicebio, mouse monoclonal 1A4) and Cy3 conjugated Goat Anti-rabbit IgG (H+L) (Servicebio) or Alexa Fluor^®^ 488-conjugated Goat Anti-mouse IgG (H+L) (Servicebio) were used for generating fluorescence staining. The stained sections were subjected and observed by microscopy (Olympus BX53, Tokyo, Japan). The average IOD of HIF-1α, CD31, and NLRP3 in five randomly selected areas for each group were calculated by using Imagepro Plus 6.0 (Media Cybernetics, Inc.).

### Western Blot Analysis of Tumor Tissue

Fresh tumor tissues were lysed with ultrasonication in RIPA cell lysis buffer (Beyotime Biotechnology, Haimen, China) supplemented with 1 mM PMSF. The lysates were clarified by centrifugation at 12,000 rpm for 15 min at 4°C. After denatured by boiling in loading buffer (Transgen Biotech, China), an equal amount of protein (20 μg) was loaded and separated on 12% glycine SDS-PAGE gel, and transferred to polyvinylidene difluoride (PVDF) membranes (0.45 μm; Millipore, Bedford, MA). CD31 (Abcam, Rabbit polyclonal to CD31), HIF-1α (Abcam, EPR16897, Rabbit mAb), and β-actin (CST, 8H10D10, Mouse mAb) were probed with specific primary antibodies and HRP-conjugated secondary anti-mouse and anti-rabbit antibodies (Jackson ImmunoResearch, West Grove, PA). The immune-complex on the membrane was visualized using an automatic chemo-luminescence image analysis system (Tanon, Shanghai, China) with HRP substrate luminol reagent and peroxide solution (Millipore).

### Statistical Analysis

All data were shown as mean and standard error of the mean (SEM). Student's *t*-tests (two-tailed) were used to assess the statistical significance of experimental results, except the survival data. Survival was assessed using the log-rank test (Mantel-Cox). All statistical analyses were performed using SAS software (ver. 8.2; SAS Institute. ^*^P < 0.05, ^**^P < 0.01, ^***^P < 0.001).

### Ethical Statement

All the animal experiments reported herein were carried out in compliance with the regulations of Chinese Academy of Medical Sciences Standing Committee on animal experiments.

## Results

### Physicochemical Characterization and Cytotoxicity of e-As_4_S_4_

**Figure 1:**
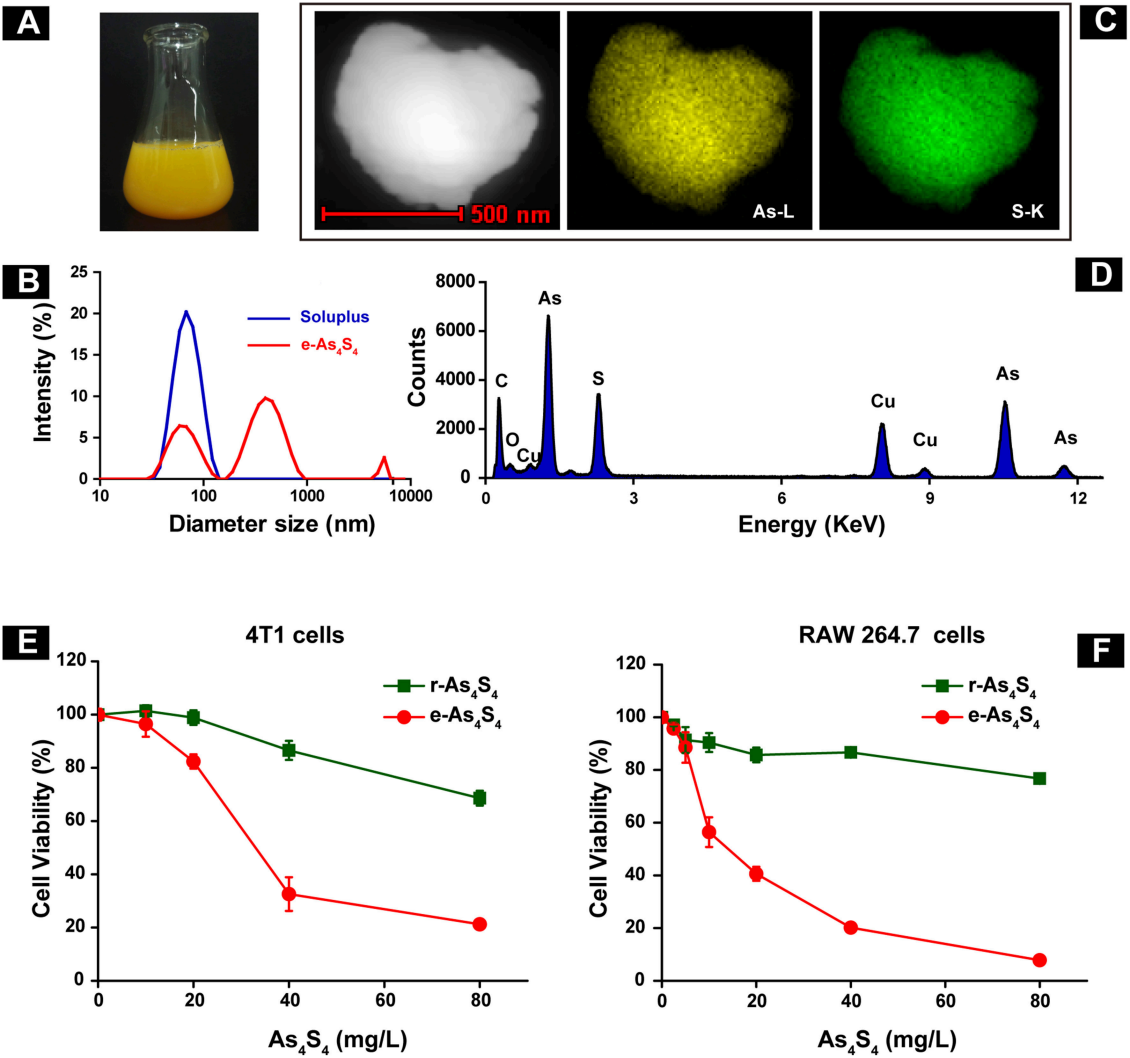
Characterization and cytotoxicity of e-As_4_S_4_. **(A)** e-As_4_S_4_ dissolved in saline. **(B)** Size distribution of e-As_4_S_4_ and Soluplus measured by DLS. **(C)** STEM images of a representative e-As_4_S_4_ nanoparticle. **(D)** EDX mapping of an e-As_4_S_4_ nanoparticle. The relative viability of the mouse breast cancer cells 4T1 **(E)** and mouse macrophages RAW 264.7 **(F)** upon the treatment of e-As_4_S_4_ and r-As_4_S_4_.

The e-As_4_S_4_ was able to dissolve in saline rapidly, forming a yellow colloid solution (Figure 1A). Results of the dynamic light scattering (DLS) measurement showed that the polymeric molecules themselves could form micelles of 67 nm in diameter when there were no nanoparticles of As_4_S_4_. There were three peaks observed for the colloid solution of e-As_4_S_4_, attributed from left to right to the polymer micelle alone, As_4_S_4_ nanoparticles encapsulated with the polymeric micelle (e-As_4_S_4_) and a small part of aggregation, respectively (Figure 1B). The colloid solution was stable for hours in a slightly mixing condition, PDI was about 0.4–0.5. The solution was acceptable for intragastric administration and for *in vitro* cellular experiments. The hydrodynamic diameter of e-As_4_S_4_ was about 700 nm, STEM observation showed the average diameter e-As_4_S_4_ was about 600 nm, and the particles were detected element arsenic (green), and sulfate (yellow) by EDX analysis (Figures 1C,D). The cytotoxicity of e-As_4_S_4_ to mouse breast cancer cell 4T1 and mouse macrophage RAW264.7 was examined by CCK-8 assay. When incubated with e-As_4_S_4_ at 10, 20, and 40 mg/L, the relative viability of 4T1 cells was 96.45, 82.36, and 32.58% (Figure 1E), while that of RAW264.7 was 56.42, 40.60, and 20.18%, respectively (Figure 1F). It should be noted that the macrophages were more sensitive to e-As_4_S_4_ than 4T1 cells.

### e-As_4_S_4_ Accumulated in Tumor Tissue and Reduced ROS in the Tumor Tissue

**Figure 2:**
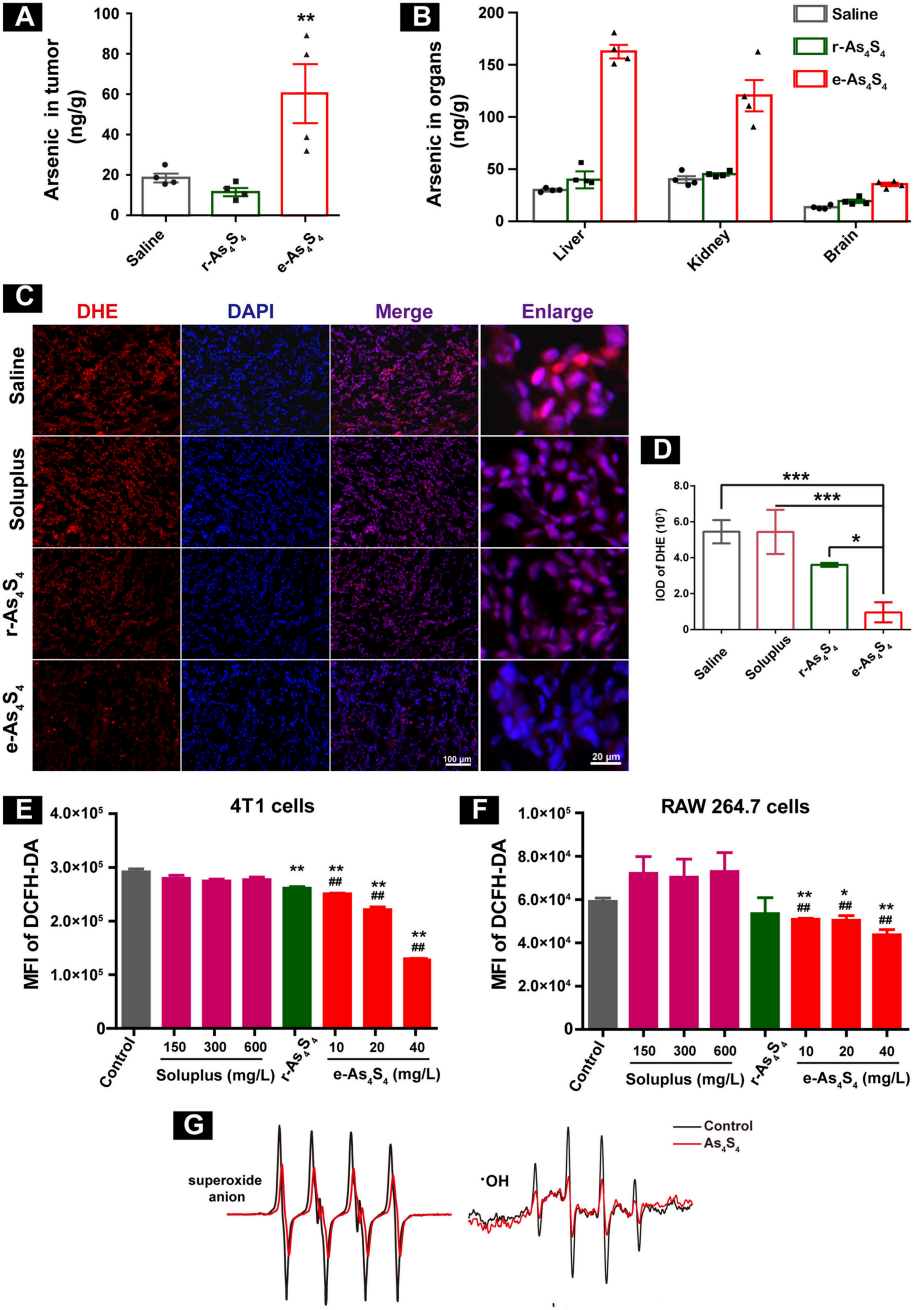
e-As_4_S_4_ accumulated in tumor tissue and reduced ROS in cells and tumor tissue. **(A)** Arsenic content in the tumor tissues. **(B)** Arsenic content in the liver, kidney and brain. **(C)** The ROS expression in the tumor tissues. **(D)** Quantification of the DHE staining. **(E,F)** Intracellular ROS of the breast cancer tumor cell 4T1 and macrophages after treated with e-As_4_S_4_ for 24 h, respectively, *p < 0.05, **p < 0.01, ***p < 0.001 vs. saline group; ##*p* < 0.01 vs. soluplus group. **(G)** ESR spectra of superoxide anion and ·OH in the absence or presence of As_4_S_4_.

In order to evaluate the distribution of arsenic in the breast cancer tumor 4T1 cell inoculation mouse model, liver, kidney, brain, and tumor mass (Figure S1) were collected after 21 days administration, followed by digestion and analysis by using AFS. Importantly, a considerable increase of arsenic was detected in the tumor mass (Figure 2A), about 80 ng/g in the e-As_4_S_4_ group, while 20 ng/g in the saline or r-As_4_S_4_ group, reaching a more than 4 fold increase. It was noticeable that the arsenic content in liver and kidney was increased in the e-As_4_S_4_ group as well, compared with that in the other two groups (Figure 2B), but few in the brain, indicating the arsenic not able to pass the blood-brain barrier.

Reactive oxygen species (ROS) homeostasis is crucial for maintaining the behavior of cancer cells (13, 14). Accumulating evidence indicated that tumor cells usually have increased ROS levels compared to normal cells, because of their accelerated metabolism and hypoxia microenvironment (15, 16), which may, in turn, affect redox-sensitive molecules such as HIF-1α and further stimulate tumor cells proliferation, migration and invasion (17, 18). When the tumor-bearing mice were treated with e-As_4_S_4_, the ROS level in the tumor mass was down-regulated detected by using DHE staining (Figure 2C), the red stainings (ROS positive) were much less intense in the group of e-As_4_S_4_ than that in the group of saline and soluplus. Compared to saline, the average IOD of DHE for e-As_4_S_4_ group was much lower than that for r-As_4_S_4_ (Figure 2D). It was interesting to see that e-As_4_S_4_ reduced ROS levels of 4T1 cells and macrophages significantly at the all tested concentration (Figures 2E,F). It could be noticed that As_4_S_4_ in its original status (r-As_4_S_4_) did not exhibit the function of reducing ROS of tumor tissues, though it decreased the intracellular ROS in the tumor cells. To validate the ability of As_4_S_4_ scavenging ROS, electron spin resonance (ESR) spectroscopy was performed in aqueous solutions. Results showed that As_4_S_4_ was able to weaken the signal of both ·OH and superoxide anion (Figure 2G), indicating that As_4_S_4_ could scavenge ·OH and superoxide anion. To address whether the lower ROS is due to higher antioxidant response or not, MnSOD expression level in the tumor tissues was investigated. Results showed that the intensity of brown-colored area decreased in e-As_4_S_4_ group (Figure S2), suggesting that e-As_4_S_4_ reduced ROS in tumor tissue, which led to the lower MnSOD.

### e-As_4_S_4_ Down-Regulated HIF-1α and CD31 in Tumor Tissue

**Figure 3:**
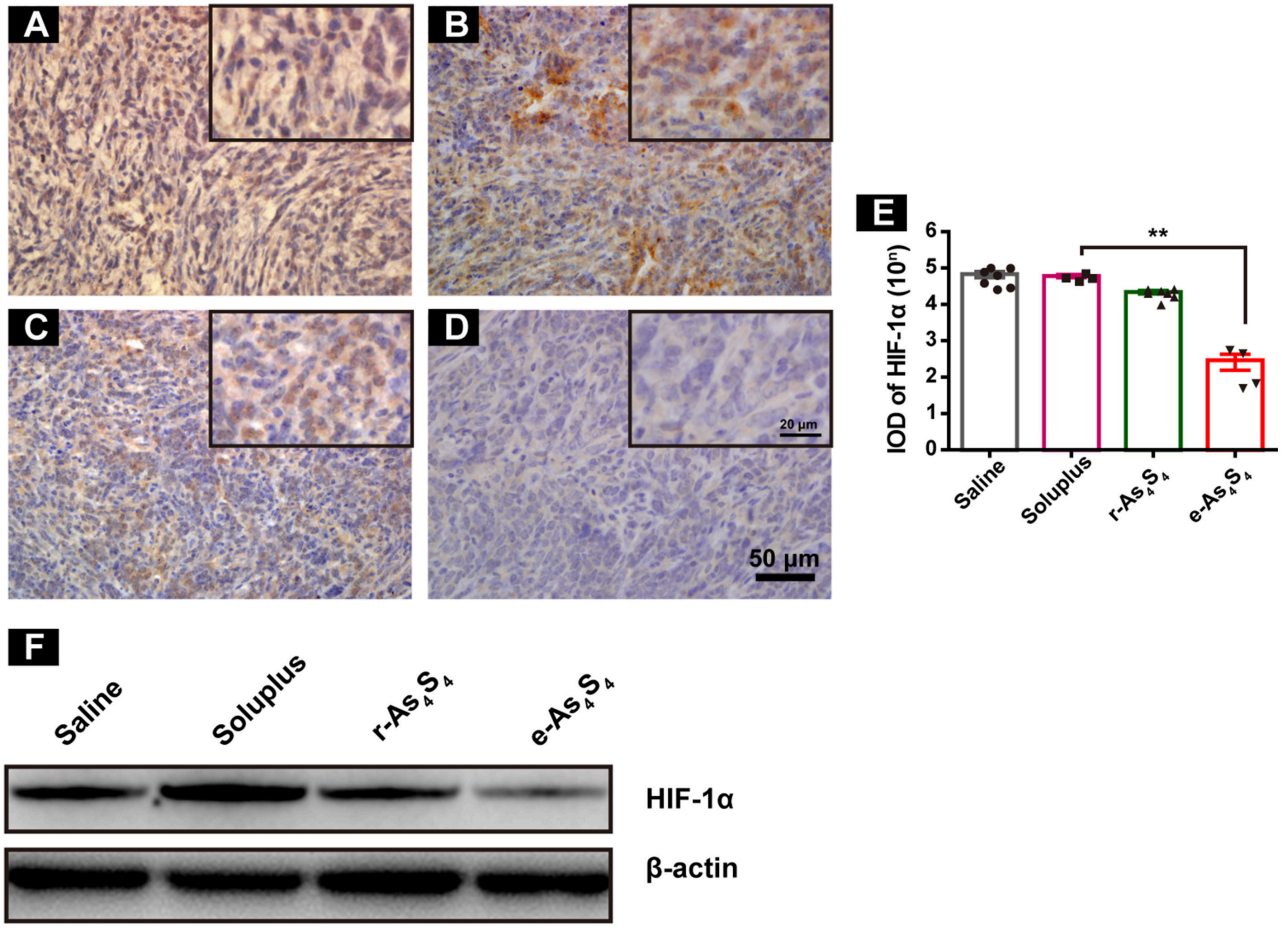
e-As_4_S_4_ down-regulated HIF-1α in tumor tissue. IHC staining of HIF-1α in tumor tissues of saline group **(A)**, soluplus group **(B)**, r-As_4_S_4_ group **(C)**, and e-As_4_S_4_ group **(D)**. Scale bar = 50 μm (whole image), scale bar = 20 μm (inserted). **(E)** IOD of HIF-1α expression of the four groups **p < 0.01. **(F)** Western blot analysis of HIF-1α for tumor tissues lysate.

The central area of the solid tumor is generally hypoxic because of the rapid growth of tumor cells (19). The hypoxic condition may enhance abnormal angiogenesis, invasion and metastasis of tumors (19–21). Hypoxia-inducible factors (HIF) are activated upon hypoxia. We next examined HIF-1α expression in the primary tumor tissues by IHC staining and western blotting. It was observed that the tumor tissue of saline and soluplus group highly expressed HIF-1α (Figures 3A,B), and r-As_4_S_4_ administration mildly down-regulated HIF-1α expression with weak brown color staining in tumor tissue (Figure 3C), while much less HIF-1α was detected in the e-As_4_S_4_ group (Figure 3D). Compared to saline, the average IOD of HIF-1α for e-As_4_S_4_ group was much lower than that for r-As_4_S_4_ (Figure 3E). Results obtained from the western blotting analysis showed that the total HIF-1α expression of tumor lysate for the e-As_4_S_4_ group was much lower than that for r-As_4_S_4_ group (Figure 3F), which was consistent with the IHC images. It was noticeable that the mRNA of Hif1a in the tumor tissue of e-As_4_S_4_ group was also decreased significantly (Figure S3), which indicated that the level of HIF-1α protein was at least partly regulated by e-As_4_S_4_ transcriptionally.

Additionally, staining for carbonic anhydrase IX (CAIX) was performed, which indicates the functional activity of HIF and indicates hypoxic areas. Results of the IHC staining showed e-As_4_S_4_ induced the down regulation of CAIX (Figure S4), which was consistent with that of HIF-1α. Expression of some downstream genes was evaluated and results showed that e-As_4_S_4_ upregulated the mRNA expression of Brca1 and downregulated the mRNA expression of Vegfa and Ldha (Figure S5).

**Figure 4:**
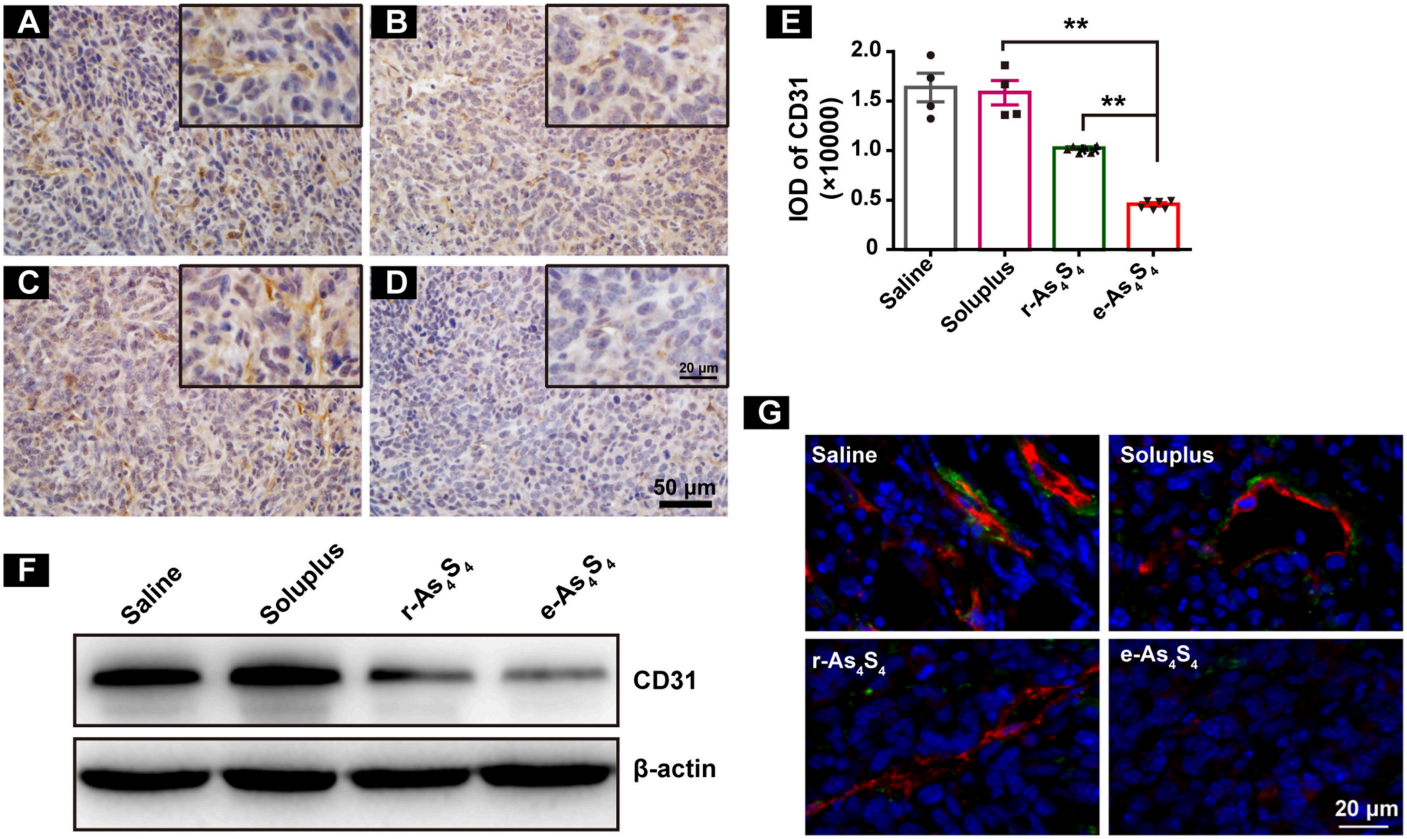
e-As_4_S_4_ down-regulated CD31 in tumor mass. IHC staining of CD31 in tumor tissues of saline **(A)**, soluplus **(B)**, r-As_4_S_4_ **(C)**, and e-As_4_S_4_ **(D)**. Scale bar = 50 μm (whole image), scale bar = 20 μm (inserted). **(E)** IOD of CD31 expression of the four groups **p < 0.01. **(F)** Western blot analysis of CD31 of tumor tissues lysate. **(G)** Combined staining of CD31 (red) and α-SMA (green) in tumor tissues. Scale bar = 20 μm.

Tumor vascular index rather than tumor size is a more reliable prognostic indicator for tumor relapse after chemotherapy (22). It has been demonstrated that a number of angiogenesis-associated genes are directly transcripted by HIF-1α (23). As a result, loss of HIF-1α decreased tumor vascularization. CD31 is an angiogenesis related marker as an indicator of cancer metastasis. It was shown that compared with the IHC staining result of saline group (Figure 4A), soluplus group (Figure 4B) and r-As_4_S_4_ group (Figure 4C), positive CD31 staining in the e-As_4_S_4_ group was the weakest one (Figure 4D), following was the r-As_4_S_4_ group. The IOD of CD31 for each group was given in Figure 4E. Results from western blotting analysis provided further evidence that the expression of CD31 in tumor mass was significantly reduced in the e-As_4_S_4_ group than that in the other groups, following was the r-As_4_S_4_ group (Figure 4F). Combined staining of CD31 and α-SMA showed that in the saline and soluplus group, there were pericyte positive endothelial structures, while in the r-As_4_S_4_ group, endothelial structures were pericyte negative, and hardly any endothelial structures could be appreciated in e-As_4_S_4_ group (Figure 4G).

### e-As_4_S_4_ Reduced NLRP3 in Tumor Tissue

**Figure 5:**
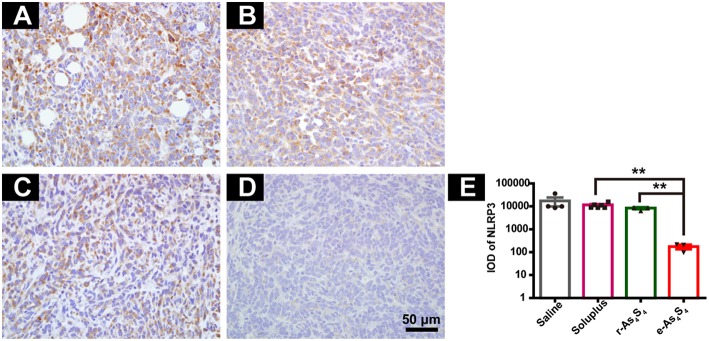
e-As_4_S_4_ reduced the expression of NLRP3 in tumor tissue. IHC staining of NLRP3 in the tumor tissue of saline **(A)**, soluplus **(B)**, r-As_4_S_4_ **(C)**, and e-As_4_S_4_ **(D)**. **(E)** IOD of NLRP3 expression of the four groups **p < 0.01. Scale bar = 50 μm.

Inflammasomes are large protein complexes, typically composed of a NOD-like receptor, caspase-1, and the adaptor protein ASC. NLRP3 is constitutively activated and leads to sustained local and systemic inflammation mediated by IL-1β in the tumor (24). Oxide stress was also closely associated with the formation and activation of NLPR3 inflammasomes (25). The IHC staining with tumor tissues showed the strong expression of NLRP3 in the saline group and soluplus group (Figures 5A,B), while r-As_4_S_4_ treatment decreased the NLRP3 expression slightly (Figure 5C). It was striking to find out that e-As_4_S_4_ significantly inhibited the NLRP3 expression, there was not appreciated positive staining of NLRP3 observed in most of the area in tumor tissues (Figure 5D). The IOD of NLRP3 positive staining of e-As_4_S_4_ were significantly lower than the other three groups (Figure 5E).

### e-As_4_S_4_ Inhibited Metastasis to Lung and Liver

**Figure 6:**
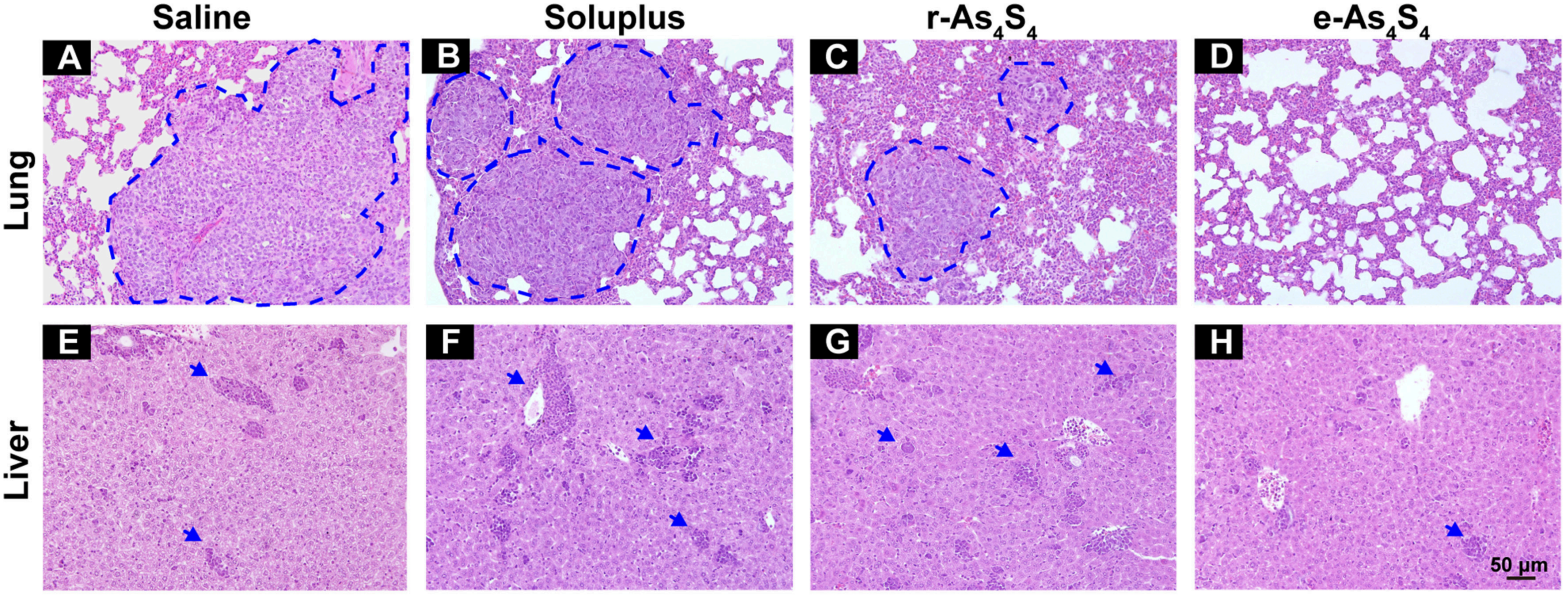
e-As_4_S_4_ inhibited metastasis to lung and liver. Representative H&E staining of lung tissue for saline **(A)**, soluplus **(B)**, r-As_4_S_4_ **(C)**, and e-As_4_S_4_ **(D)**; liver tissue in saline **(E)**, soluplus **(F)**, r-As_4_S_4_ **(G)**, and e-As_4_S_4_ **(H)**. The metastasis nodules in the lung tissues were circled by blue dotted lines and the metastasis nodules in the livers were pointed by blue arrowheads. Scale bar = 50 μm.

4T1 cells are highly metastatic (26), and the 4T1 breast cancer model is a standard one equivalent to the fourth stage of human metastatic breast cancer as it shares many common characteristics with human mammary cancer with spontaneous pulmonary metastasis from a palpable tumor (27, 28). After 3 week treatment with saline, soluplus, r-As_4_S_4_ or e-As_4_S_4_, five mice in each group were sacrificed, and H&E staining were performed to detect metastasis in lungs and livers (Figure 6). Large metastasis nodules were observed in the lung of saline and soluplus group (blue dotted line circled, Figures 6A,B), and r-As_4_S_4_ exhibited mild effect of inhibiting metastasis in lung (Figure 6C); strikingly, no obvious metastasis nodules were observed in the lung of the e-As_4_S_4_ group (Figure 6D), indicating that the oral administration of e-As_4_S_4_ led to a significant inhibition of metastasis modules in lungs. In addition, there were metastasis nodules distributing in the liver cortex and close to the portal vein of mice in the saline group (Figure 6E), while the metastasis in livers was strongly inhibited by e-As_4_S_4_, showing less and smaller metastasis nodules (Figure 6H).

### e-As_4_S_4_ Prolonged the Survival of Breast Cancer Mice

**Figure 7:**
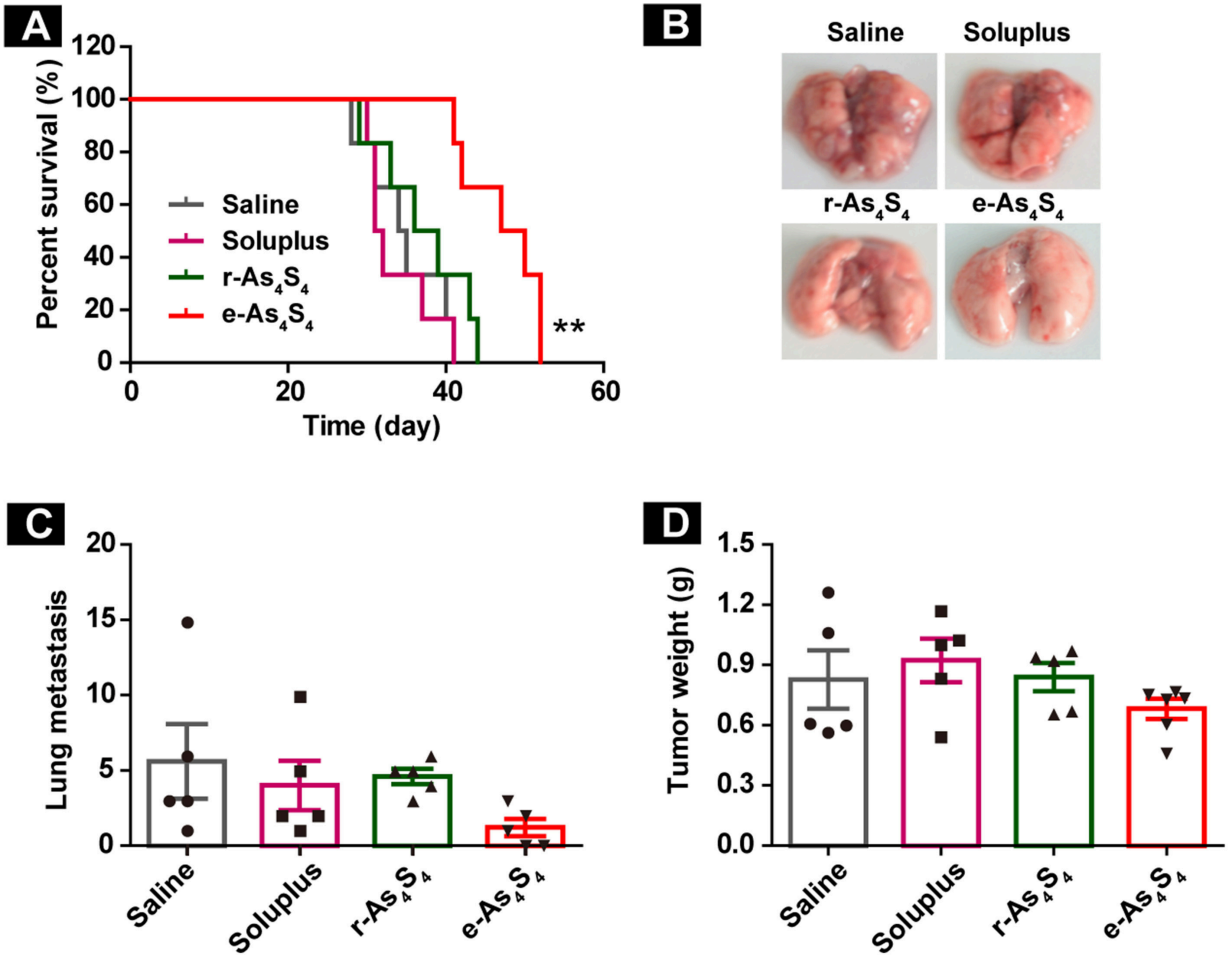
e-As_4_S_4_ prolonged the survival of breast cancer mice. **(A)** Survival curve of tumor-bearing mice of saline, soluplus, r-As_4_S_4_, and e-As_4_S_4_ **p < 0.01. **(B)** Representative gross observation of lung metastasis. **(C)** Lung metastasis nodules of the four groups after 21-day treatment. **(D)** Tumor weight after the 21-day treatment.

The therapeutic efficacy of e-As_4_S_4_ for breast cancer was further investigated *in vivo* using the 4T1 breast cancer mice. It was observed that both r-As_4_S_4_ and e-As_4_S_4_ treatments prolonged the survival of breast cancer mice compared with saline or soluplus treatment (Figure 7A). The median survival was 50 days for mice treated with e-As_4_S_4_ and 38 days for mice with r-As_4_S_4_, while that was about 30 days for the saline group. Agreed to the histological results, the 3-week treatment of e-As_4_S_4_ led to the significant decrease in the number of metastatic foci observed on the lung surface (Figures 7B,C), though e-As_4_S_4_ treatment had no significant influence on primary tumor weight (Figure 7D), suggesting the prolonged survival was attributable to the inhibition of metastasis by e-As_4_S_4_.

## Discussion

Hot-melt extrusion was one powerful technique that has been applied to improve dissolution behavior of poorly soluble organic compounds; however, the application in mineral drugs is not adequate. In this study, Soluplus was applied as a polymeric carrier for dissolution enhancement of As_4_S_4_ using hot-melt extrusion because the pure As_4_S_4_ is poorly soluble in water and acidic aqueous solution. By the co-extrusion technique, the crystal of As_4_S_4_ was squeezed into nanoparticles by the two screws, and meanwhile those nanoparticles were encapsulated by the melting soluplus. The resulting formulation was able to dissolve in saline to form a colloid solution, by this way the bioavailability of As_4_S_4_ as well as the *in vivo* efficacy was increased. In the current study, the dose of 180 mg/Kg/day was effective in the tumor bearing mice, which could be converted to 15 mg/Kg/day for human. It was reported that in clinic orally administration of As_4_S_4_ at a dosage of 50 mg/kg/day was effective and safe in both remission induction and maintenance therapy in patients with APL (8). Therefore, the envisaged dose of e-As_4_S_4_ would be much lower than the current clinical dose.

In the progress of a solid tumor, the chaotic proliferation of transformed cells always resulted in hypoxia region in the tumor tissue due to an imbalance between oxygen delivery and oxygen consumption (19). Hypoxic is the typical characteristics of the tumor microenvironment, especially for the highly metastatic one (29, 30). Breast cancer, like other solid malignancies, is highly hypoxic (31). It is estimated that 40% of all breast cancers and 50% of locally advanced breast cancer have hypoxic regions (32). It is well documented that hypoxia activates HIF signaling, which serves as master regulators of breast cancer metastasis by activating the transcription of genes encoding proteins that participate in multiple steps of the metastatic process in TNBC (33, 34).

In the present study, we observed the associations between the antitumor activity of e-As_4_S_4_, reduced level of ROS and HIF-1α down regulation, suggesting it a promising strategy of regulating ROS to correct the hypoxia associated behaviors in the solid tumor. It is well documented that ROS can regulate HIF-1α through both transcriptional and post-translation level (35, 36), therefore we inferred that the resulting down regulation of HIF-1α by e-As_4_S_4_ was at least partly regulated transcriptionally, which ultimately led to the decrease of Hif1a gene expression, but whether the reduced ROS regulated HIF-1α destablization was not elucidated yet in the present study. Additionally, more than one factors are involved in HIF-1α destabilization in tumors. For instance, HIF-1α stabilization may depend on the state of respiratory complex I and mitochondrial respiration (36). These indicated that comprehensive demonstration for mechanisms on the relationship between HIF-1α and ROS is very necessary in further investigations.

In the current study, we showed that e-As_4_S_4_ could accumulate in the tumor tissue due to the reduced particle size and the encapsulation by the hydrophilic polymer. Arsenic of e-As_4_S_4_ nanoparticle is present in the form As(II). It has been reported that the As(II) is unstable in water and can be oxidized to As(III) before dissolving in aqueous solution (37). Therefore, it is reasonable to confer that As(II) of e-As_4_S_4_ nanoparticle would be oxidized to As(III) by ROS generated in the tumor microenvironment, which resulted in the consumption of ROS in the tumor tissue, and the reaction would be in high effectiveness because the nanoparticles had high specific surface. The ROS consumption and cytotoxicity of e-As_4_S_4_ contributed together to build a less aggressive microenvironment for the tumor cells, evidenced by the reduction of CD31 (Figure 4) (23). In addition, ROS also play great parts in tumor-associated inflammation through activation of inflammasome (25). It was noticeable that e-As_4_S_4_ eliminated NLRP3 inflammasomes significantly in tumor tissue (Figure 5), indicating that e-As_4_S_4_ could change the inflammatory microenvironment to some extents in the tumor tissue.

Although there are reports that some dietary antioxidants inhibited tumor initiation and progression, clinical studies have failed to show consistent data demonstrating the protective effects of these substances on cancer incidence (38). Therefore, we would suggest the role of scavenging ROS is one way for the therapeutic effect of e-As_4_S_4_, and the inhibitory effect on the viability of macrophages as well as tumor cells (Figures 1E,F) might be involved in other mechanisms. Macrophages infiltration is another contributor to tumor-associated inflammation. Macrophages participate in the promotion of angiogenesis and tumor growth and progression in both animal models and patients with cancer, with their presence associated with poor clinical outcomes (24). Therefore, mechanisms from different views are worth investigating in the future.

Taken together, we demonstrated that the effects of reducing intracellular ROS, down regulating HIF-1α and inhibiting inflammatory microenvironment for e-As_4_S_4_ contributed to the metastasis inhibition in the TNBC mouse model, therefore prolonged the survival of the mice, which indicated its attractive potential in treating TNBC.

## Conclusion

It was showed that e-As_4_S_4_ could alleviate the metastatic features of aggressive breast cancers through correcting the tumor microenvironment, which prolonged the survival of tumor-bearing mice, showing its important therapeutic implications to the anti-metastasis therapy for breast cancer.

## Ethics Statement

Six-week-old, female BALB/c mice were bred at the Experimental Animal Center at the Institute of Basic Medical Sciences, Chinese Academy of Medical Sciences (Beijing, China) under specific pathogen-free conditions. All the animal experiments reported herein were carried out in compliance with the regulations of Chinese Academy of Medical Sciences Standing Committee on animal experiments.

## Author Contributions

HX and JM designed experiments, discussed all results with authors and revised the manuscript. JM and TWa performed the main experiments, made data analysis, prepared figures and wrote the main manuscript. CW performed part of the survival experiment on mice. TWe performed the ESR measurement, reviewed the manuscript and discussed results. MJ performed the cytotoxicity and ROS assay on 4T1 cells and RAW264.7 cells, SH and YG performed the Western Blot experiment. LX took photos of the H&E staining. JL reviewed the manuscript and discussed results.

### Conflict of Interest Statement

The authors declare that the research was conducted in the absence of any commercial or financial relationships that could be construed as a potential conflict of interest.
