# Supplementary material for: Inhibition of Murine Breast Cancer Metastases by Hydrophilic As4S4 Nanoparticles Is Associated With Decreased ROS and HIF-1α Downregulation
Source: Front Oncol. 2019 Apr 26;9:333. doi: 10.3389/fonc.2019.00333 (PMC6498897; doi:10.3389/fonc.2019.00333)
Supplement: Supplementary file 1 [file Data_Sheet_1.pdf]

Supporting information

Inhibition of murine breast cancer metastases by hydrophilic As_4S_4 nanoparticles is associated with decreased ROS and HIF-1α downregulation

Tao Wang[a], Jie Meng[a*], Chuan Wang[a], Tao Wen[a], Mengfan Jia[a], Yangyang Ge[a], Lifei Xie[a], Suisui Hao[a], Jian Liu[a], Haiyan Xu[a*]

a. Institute of Basic Medical Sciences, Chinese Academy of Medical Sciences & Peking Union Medical College. 100005, Beijing, China

***Main corresponding author:** Haiyan Xu
Address: 5 Dong Dan San Tiao, Beijing 100005, China;
E-mail: xuhy@pumc.edu.cn
Tel: (+86)10 69156437

***Additional corresponding author:** Jie Meng
Address: 5 Dong Dan San Tiao, Beijing 100005, China;
E-mail: mengjie@ibms.pumc.edu.cn
Tel: (+86)10 65135502

Results

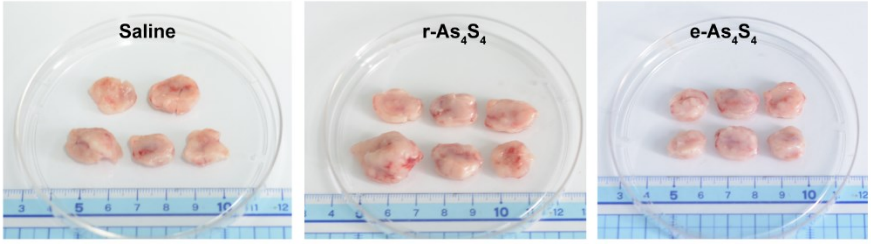

Figure S1. Raw pictures of tumor tissues for arsenic content quantification.

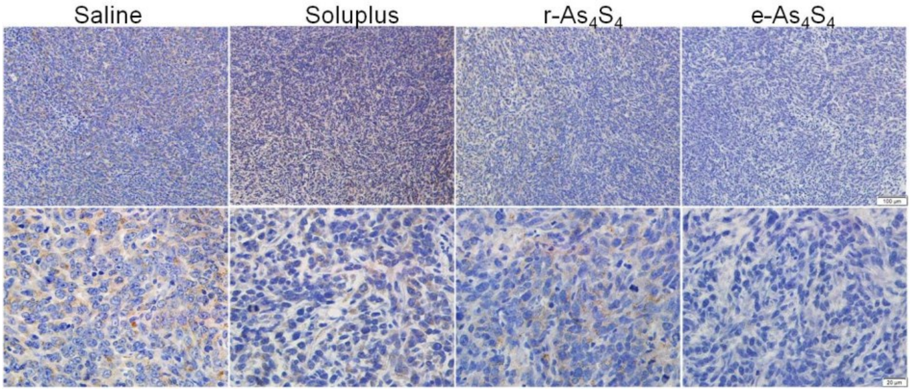

Figure S2. e-As_4S_4 downregulated MnSOD expression in tumor mass. Low magnifciation of images were showed in the up row (Scale bar=100 μm) and larger magnifciation of images in the bottom row (Scale bar=20 μm).

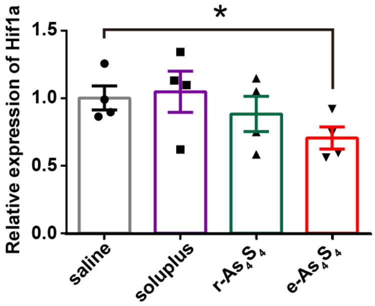

Figure S3. e-As_4S_4 decreased the mRNA expression of Hif1a in tumor tissues.
* $p<0.05$.

Method for the quantitative Real-Time PCR of tumor tissues:

Frozen tumor tissues were ground with TRIzol reagent (Sigma-Aldrich). Total RNA was extracted and reverse transcribed into cDNA via reverse transcription PCR. Quantitative real-time PCR was performed using SRBY probe (Takara Bio, Shiga, Japan) and GAPDH was used as an internal control. The following primers were used:

Hif1a forward primer 5' ACCTTCATCGGAAACTCCAAAG3'
Hif1a reverse primer 5' CTGTTAGGCTGGGAAAAGTTAGG3'
Gapdh forward primer 5'AGGTCGGTGTGAACGGATTTG3'
Gapdh reverse primer 5'TGTAGACCATGTAGTTGAGGTCA3'.

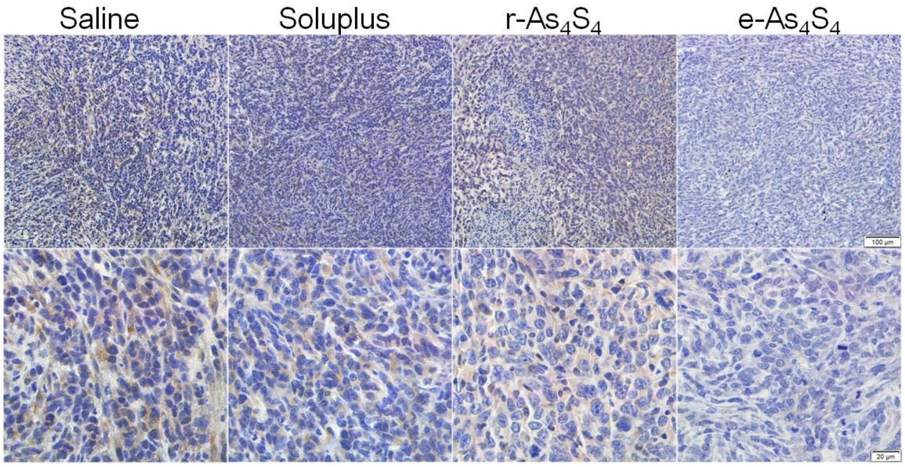

Figure S4. e-As_4S_4 downregulated CAIX expression in tumor mass. Low magnifciation of images were showed in the up row (Scale bar=100 μm) and larger magnifciation of images in the bottom row (Scale bar=20 μm).

Method for the immunohistochemical analysis of MnSOD and CAIX:
The slides were deparaffinized and antigen retrieval was then performed using microwave oven in EDTA, pH=8.0 (Servicebio, Wuhan, China). Primary antibodies of MnSOD (GenTex, Rabbit IgG polyclonal), CAIX (GeneTex, Rabbit Polyclonal) were applied overnight before HRP-labeled Goat Anti-Rabbit IgG (H+L) (Servicebio) incubation for 50 minutes at room temperature. DAB was used as chromogens and slides were counterstained with haematoxylin before mounting.

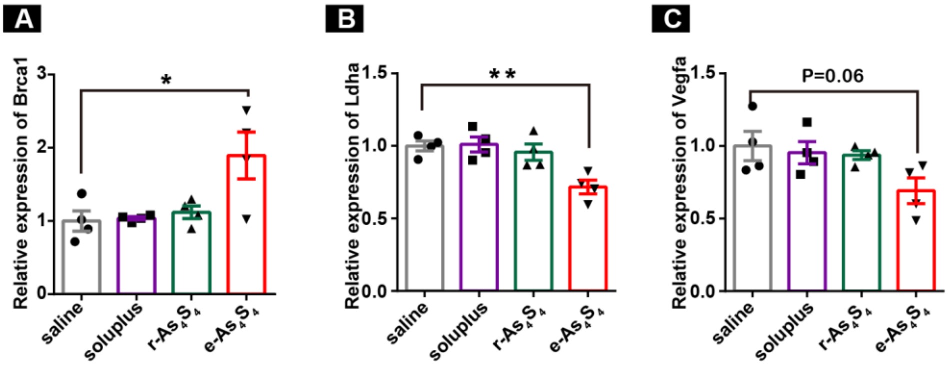

Figure S5. e-As_4S_4 increased the mRNA expression of Brca1 (A) while decreased that of Ldha (B) and Vegfa (C). * $p<0.05$, ** $p<0.01$.

Method for the quantitative Real-Time PCR of tumor tissues:

Frozen tumor tissues were ground with TRIzol reagent (Sigma-Aldrich). Total

RNA was extracted and reverse transcribed into cDNA via reverse transcription PCR. Quantitative real-time PCR was performed using SRBY probe (Takara Bio, Shiga, Japan) and GAPDH was used as an internal control. The following primers were used:

Vegfa forward primer 5'GCACATAGAGAGAATGAGCTTCC3',
Vegfa reverse primer 5'CTCCGCTCTGAACAAGGCT3',
Ldha forward primer 5'TGTCTCCAGCAAAGACTACTGT3',
Ldha reverse primer 5'GACTGTACTTGACAATGTTGGGA3',
Brca1 forward primer 5'CGAATCTGAGTCCCCTAAAGAGC3',
Brca1 reverse primer 5'AAGCAACTTGACCTTGGGGTA3'
Gapdh forward primer 5'AGGTCGGTGTGAACGGATTTG3'
Gapdh reverse primer 5'TGTAGACCATGTAGTTGAGGTCA3'.
